# Genetic and Physical Mapping of Candidate Genes for Resistance to *Fusarium oxysporum* f.sp. *tracheiphilum* Race 3 in Cowpea [*Vigna unguiculata* (L.) Walp]

**DOI:** 10.1371/journal.pone.0041600

**Published:** 2012-07-31

**Authors:** Marti Pottorff, Steve Wanamaker, Yaqin Q. Ma, Jeffrey D. Ehlers, Philip A. Roberts, Timothy J. Close

**Affiliations:** 1 Department of Botany & Plant Sciences, University of California Riverside, Riverside California, United States of America; 2 Bill & Melinda Gates Foundation, Seattle, Washington, United States of America; 3 Department of Nematology, University of California Riverside, Riverside, California, United States of America; Virginia Tech, United States of America

## Abstract

*Fusarium oxysporum* f.sp. *tracheiphilum* (Fot) is a soil-borne fungal pathogen that causes vascular wilt disease in cowpea. Fot race 3 is one of the major pathogens affecting cowpea production in California. Identification of Fot race 3 resistance determinants will expedite delivery of improved cultivars by replacing time-consuming phenotypic screening with selection based on perfect markers, thereby generating successful cultivars in a shorter time period. Resistance to Fot race 3 was studied in the RIL population California Blackeye 27 (resistant) x 24-125B-1 (susceptible). Biparental mapping identified a Fot race 3 resistance locus, *Fot3-1,* which spanned 3.56 cM on linkage group one of the CB27 x 24-125B-1 genetic map. A marker-trait association narrowed the resistance locus to a 1.2 cM region and identified SNP marker 1_1107 as co-segregating with *Fot3-1* resistance. Macro and microsynteny was observed for the *Fot3-1* locus region in *Glycine max* where six disease resistance genes were observed in the two syntenic regions of soybean chromosomes 9 and 15. *Fot3-1* was identified on the cowpea physical map on BAC clone CH093L18, spanning approximately 208,868 bp on BAC contig250. The *Fot3-1* locus was narrowed to 0.5 cM distance on the cowpea genetic map linkage group 6, flanked by SNP markers 1_0860 and 1_1107. BAC clone CH093L18 was sequenced and four cowpea sequences with similarity to leucine-rich repeat serine/threonine protein kinases were identified and are cowpea candidate genes for the *Fot3-1* locus. This study has shown how readily candidate genes can be identified for simply inherited agronomic traits when appropriate genetic stocks and integrated genomic resources are available. High co-linearity between cowpea and soybean genomes illustrated that utilizing synteny can transfer knowledge from a reference legume to legumes with less complete genomic resources. Identification of Fot race 3 resistance genes will enable transfer into high yielding cowpea varieties using marker-assisted selection (MAS).

## Introduction

*Fusarium oxysporum* f.sp. *tracheiphilum* (Fot) is a soil-borne fungal pathogen which causes vascular wilt disease in cowpea [1]. Fusarium wilt disease can be problematic wherever cowpea is grown. Incidents of Fusarium wilt have been reported in the North Western Territory of Australia, northeastern parts of Brazil as well as Nigeria [2], [3], [4]. Fusarium wilt is especially problematic in cowpea production regions within the United States including the southeastern United States and the Central Valley of California [5]. The pathogen invades the vascular tissue via the root system, causing wilting and chlorosis of the leaves and sometimes stunting of the entire plant. Broad patches of infected cowpea plants are observed in fields infested with this pathogen. The outward symptoms typically become evident at the seedling stage or during flowering and early pod development, resulting in high mortality in the affected areas with significant overall yield loss.

Breeding to develop Fusarium-resistant cowpea cultivars began in the 1930’s in California after the disease was recognized [6]. Several races of Fot have evolved, races 1, 2, 3, and 4, which are identified according to differential interactions on several cowpea genotypes [5], [6], [7]. Currently, Fot race 3 is the predominant and most widely distributed race [7]. Alternative disease management practices such as applications of fungicides are not economically feasible and there are possible health and environmental concerns with such approaches. Host plant resistance is a proven strategy for managing Fusarium wilt disease in cowpea, and in infested production areas all new varieties must have resistance to race 3 and preferably to race 4 as well. Several successful cultivars have been bred specifically for their resistance to Fot race 3 combined with preferred agronomic traits, for example, California Blackeye 27, California Blackeye 46 and recently released California Blackeye 50 [8], [9]. These cultivars were developed using conventional breeding approaches that rely on phenotypic assessments as a basis for selection. For Fot race 3 resistance, several rounds of phenotypic selection are typically needed to identify and confirm putative resistant individuals during the breeding process. Marker-assisted selection (MAS) reduces the time and effort needed for the phenotypic evaluation portion of the breeding process, but may not be fully efficient due to recombination between the trait determinant and marker, proportional to their cM distance. Less than full linkage between the trait and marker will result in some individuals being misclassified during the selection process. Identification of the genetic determinants for Fot race 3 resistance will enable development of gene-based ‘perfect markers’ that will improve the efficiency of transferring resistance into elite varieties.

Molecular genetic and genomic resources have been developed for cowpea with an objective of enhancing breeding programs for improving cowpea varieties for the United States, India, Brazil and numerous countries in Africa and Asia. These integrated genomic resources include a 1536 SNP genotyping platform, an EST-derived SNP cowpea consensus genetic map, known syntenic relationships between cowpea, *Medicago truncatula*, *Glycine max* and *Arabidopsis thaliana*, and a cowpea EST sequence collection housed in HarvEST:Cowpea database (http://harvest.ucr.edu) [10]. A cowpea physical map anchored partially to the cowpea consensus genetic map using the same SNP markers is also available (http://phymap.ucdavis.edu/cowpea). In addition, >500 cowpea accessions have been SNP genotyped (UCR cowpea group, unpublished data) and a first draft of the cowpea genome, vs.0.02, has been assembled (www.harvest-blast.org). These resources will enable dissection of the underlying genetic component(s) of this trait, which will facilitate cultivar improvement using marker-assisted breeding.

The goal of this study was to identify and precisely map Fot race 3 resistance determinants in the cowpea genome. Outcomes of this study are to develop molecular markers closely linked to the *Fot3-1* resistance gene which will support breeding efforts to produce Fusarium-resistant cowpea varieties. In addition, candidate genes for the *Fot3-1* locus were identified, enabling opportunities for functional analysis which can benefit Fusarium studies in other crop plants.

**Figure 1:**
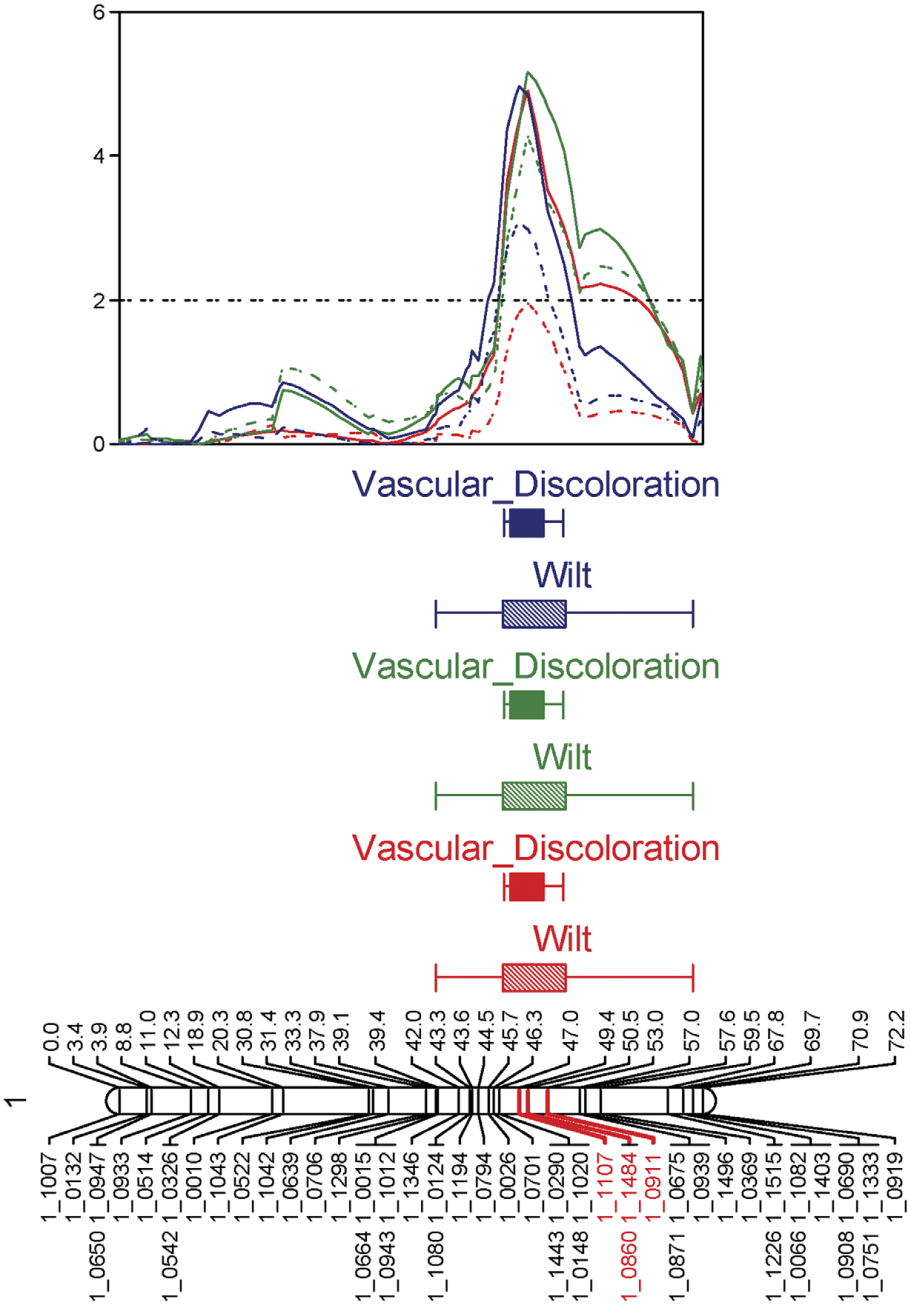
Resistance to *Fusarium oxysporum* f.sp. *tracheiphilum* race 3 in the CB27 x 24-125B-1 population. The *Fot3-1* locus (Interval Mapping analysis shown) spanned approximately 12.5 cM on the CB27 x 24-125B-1 genetic map, linkage group 1. The 2007 experiment LOD scores are plotted in red; the 2009a experiment is plotted in green and 2009b experiment is plotted in blue. Solid colored lines indicate the vascular discoloration phenotype and the wilting/stunting phenotype is depicted by broken colored lines. SNP markers 1_1107, 1_0860, 1_1484 and 1_0911 which were the most significant markers over the three experiments are highlighted in red on the linkage group. The LOD significance threshold of 2.0 is indicated by a dashed horizontal line on the graph.

## Results

Interval mapping analysis of three experimental datasets from the CB27 × 24-125B-1 population identified one major locus for Fot race 3 resistance. The locus spanned 3.6 cM, from 49.4 cM to 53.0 cM on linkage group 1 of the CB27 × 24-125B-1 genetic map (Figure 1, Table 1). Of the two disease phenotypes, vascular discoloration symptoms resulted in higher LOD scores and explained a higher percent variation in phenotype than the wilting/stunting phenotype (Table 2). The wilting/stunting phenotype proved to be more sensitive to environmental variation than the vascular discoloration phenotype, however, it was still a good criterion for measuring disease resistance to Fusarium. SNP markers 1_1107, 1_0860, 1_1484 and 1_0911 were consistently the most significant linked markers over all three experiments based on six mapping results (Table 2). For two experiments, markers 1_0860 and 1_1484, which are in the same marker bin, accounted for the highest percent phenotypic variance for the vascular discoloration phenotype, 25.2% (LOD 4.91) and 27.3% (LOD 5.16), respectively (Table 2). Marker 1_1107 had the highest association with the vascular discoloration phenotype in the third experiment, accounting for 27.8% of the phenotypic variance (LOD 4.97) (Table 2). Henceforth, the Fot race 3 resistance locus will be referred to as *Fot3-1*.

**Table 1:** *Fot3-1* locus in the CB27 x 24-125B-1 genetic map, cowpea consensus genetic map and cowpea physical map.

CB27 x 24-125B-1 genetic map			Cowpea consensus genetic map			Cowpea physical map	
LG	cM	SNP	LG	cM	SNP	Contig	BAC clone(s)
1	52.98	1_0911	6	15.43	1_0911	1117	CM012O18
		N/A	6	16.51	1_0830	N/A	
		N/A	6	16.88	1_1381	771	CH001O04
		N/A	6	17.14	1_0895	250	CH046G19
		N/A	6	17.14	1_1077	250	CM002B24, CM015O07
		N/A	6	17.14	1_1363	250	CM015O07, CH045I01
		N/A	6	17.40	1_0897	250	CH045I01, CM002B24, CM015O07
1	50.49	1_0860	6	17.82	1_0860	250	CH076D23, CH093L18
1	50.49	1_1484	6	17.88	1_1484	N/A	
1	49.42	1_1107	6	18.31	1_1107	250	CM001C09, CM051M10
		N/A	6	19.04	1_0704	250	CM054B04, CH051M10

SNP markers are aligned in the order designated by the cowpea consensus genetic map.

**Table 2:** Bi-parental mapping of *Fot3-1* in the CB27 x 24-125B-1 population.

Experiment	Statistical analysis	Phenotype	1_1107	1_0860	1_1484	1_0911
2007	IM LOD	Wilting/Stunting	1.83	1.95	1.95	1.57
	IM R^2^	Wilting/Stunting	10.2	10.7	10.7	8.8
	IM LOD	Vascular Discoloration	4.49	4.91	4.91	3.52
	IM R^2^	Vascular Discoloration	23.2	25.2	25.2	18.7
	Kruskal-Wallis test statistic	Wilting/Stunting	6.18	6.22	6.22	5.52
	Kruskal-Wallis p-value	Wilting/Stunting	0.05	0.05	0.05	0.05
	Kruskal-Wallis test statistic	Vascular Discoloration	29.09	32.42	32.42	23.08
	Kruskal-Wallis p-value	Vascular Discoloration	0.0001	0.0001	0.0001	0.0001
2009a	IM LOD	Wilting/Stunting	3.7	4.26	4.26	3.37
	IM R^2^	Wilting/Stunting	20.2	22.7	22.7	18.4
	IM LOD	Vascular Discoloration	4.44	5.16	5.16	4.67
	IM R^2^	Vascular Discoloration	24.2	27.3	27.3	24.9
	Kruskal-Wallis test statistic	Wilting/Stunting	10.23	12.87	12.87	9.24
	Kruskal-Wallis p-value	Wilting/Stunting	0.005	0.0005	0.0005	0.005
	Kruskal-Wallis test statistic	Vascular Discoloration	12.747	15.97	15.97	14.54
	Kruskal-Wallis p-value	Vascular Discoloration	0.0005	0.0001	0.0001	0.0005
2009b	IM LOD	Wilting/Stunting	3.09	2.98	2.98	2.06
	IM R^2^	Wilting/Stunting	18.4	17.7	17.7	12.6
	IM LOD	Vascular Discoloration	4.97	4.85	4.85	3.23
	IM R^2^	Vascular Discoloration	27.8	27	27	18.9
	Kruskal-Wallis test statistic	Wilting/Stunting	13.33	12.13	12.13	8.37
	Kruskal-Wallis p-value	Wilting/Stunting	0.0005	0.0005	0.0005	0.005
	Kruskal-Wallis test statistic	Vascular Discoloration	24.19	22.63	22.63	16.03
	Kruskal-Wallis p-value	Vascular Discoloration	0.0001	0.0001	0.0001	0.0001

IM  =  Interval Mapping analysis.

The corresponding location of *Fot3-1* was positioned on the cowpea consensus genetic map using the highly significant markers from the biparental mapping study. *Fot3-1* spanned 15.4 cM to 18.3 cM on linkage group 6 of the cowpea consensus genetic map (Table 1).

**Figure 2:**
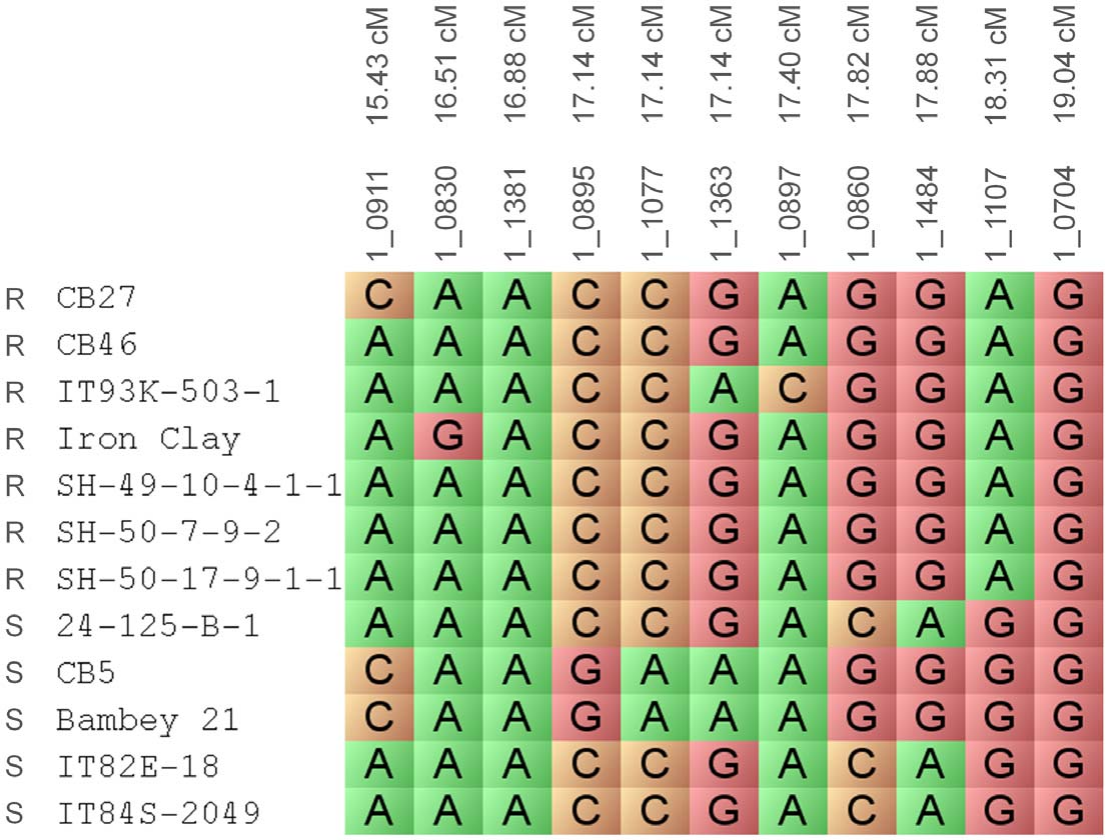
Marker-trait association of the *Fot3-1* locus. The *Fot3-1* locus on the cowpea consensus genetic map is depicted along with twelve cowpea genotypes which differ in their response to *Fusarium oxysporum* f.sp. *tracheiphilum* (Fot) race 3. “R” indicates a resistant genotype to Fot race 3 and “S” indicates a susceptible genotype to Fot race 3. SNP marker 1_1107 (18.3 cM) alleles co-segregated with the resistant and susceptible genotypes along with the corresponding disease phenotype. The adenine nucleotide is the resistant allele which is color-coded green while the susceptible allele is the guanine nucleotide which is color-coded red.

A marker-trait association panel of known Fot race 3 resistant and susceptible genotypes was used to further narrow the *Fot3-1* locus on the cowpea consensus genetic map. Genotypic data comprised of SNPs, marker loci, cowpea varieties and lines were visualized using Flapjack software (Figure 2) [11]. CB27, CB46, Iron Clay, SH49-10-4-1-1, SH50-17-9-1-1 (also known as California Blackeye No. 50), SH50-7-9-2 and West African genotype IT93K-503-1 are resistant to Fot race 3. Genotypes, 24-125B-1, CB5, Bambey 21, IT82E-18/Big Buff, and IT84S-2049 are susceptible to Fot race 3. Markers in the *Fot3-1* locus on the cowpea consensus genetic map were examined with the twelve cowpea genotypes to associate an allele with the response to Fot race 3; resistance or susceptibility. SNP marker 1_1107, which was highly significant in the biparental mapping studies, was the only marker with alleles that co-segregated perfectly with a corresponding resistant or susceptible phenotype (Figure 2). The resistant genotype at this locus is associated with the adenine nucleotide which is color-coded green in Figure 2. The susceptible genotype was associated with the guanine nucleotide which is color-coded red in Figure 2. SNP marker 1_1107 was derived from the cowpea P12 assembly unigene 12265 position 693, which was annotated as a cysteine desulfurase and can be viewed in HarvEST:Cowpea (File S1) (http://harvest.ucr.edu). The marker-trait association narrowed the *Fot3-1* locus to a 1.2 cM region and was defined by flanking SNP markers 1_1484 and 1_0704 (Figure 2).

**Figure 3:**
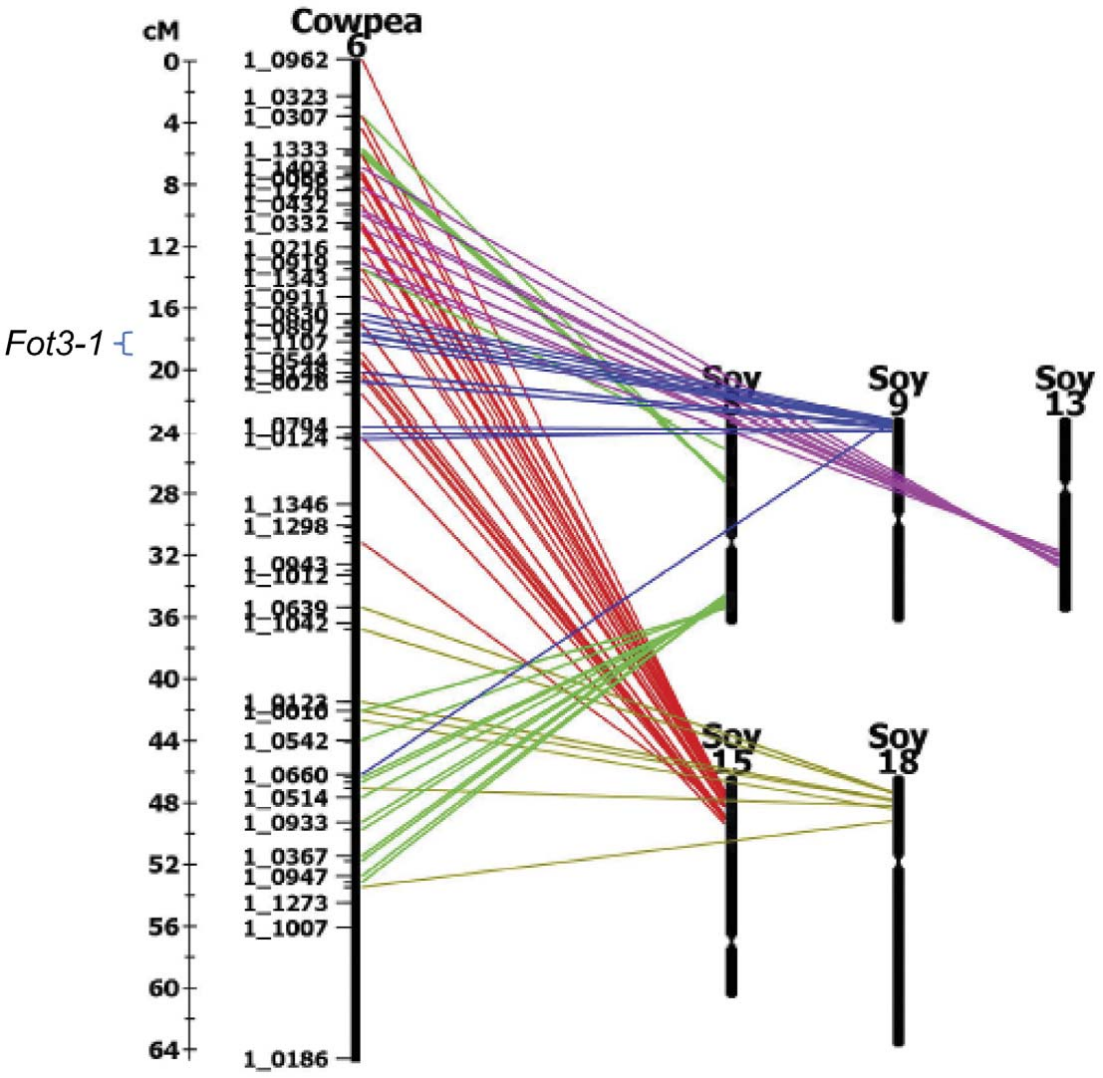
Synteny of *Fot3-1* locus with *Glycine max*. Synteny was examined for the *Fot3-1* locus between cowpea and *G. max* using EST-derived SNP markers previously BLASTed and aligned to the sequenced genome. The *Fot3-1* locus on the cowpea consensus genetic map, linkage group 6 (17.88 cM to 19.04 cM), was determined to be syntenic with soybean chromosomes 9 and 15. The *Fot3-1* syntenic locus in soybean chromosome 9 extended from soybean locus Glyma09g02100 to Glyma09g02560, where two disease resistance genes, Glyma09g02210 and Glyma09g02420, were observed. The *Fot3-1* syntenic locus in soybean chromosome 15 extended from soybean locus Glyma15g12830 to Glyma15g13470 where four disease resistance genes were observed, Glyma15g13100, Glyma15g13290, Glyma15g13300 and Glyma15g13310. The syntenic map was drawn using HarvEST:Cowpea database (http://harvest.ucr.edu) using a cut-off e-score value of −10 and a minimum number of 13 lines drawn per linkage group.

The cowpea region carrying the *Fot3-1* locus was compared with the soybean genome using HarvEST: Cowpea to determine if the gene order was conserved between species. High co-linearity with the *Fot3-1* region in any of the sequenced genomes may enable identification of candidate genes. The *Fot3-1* region was found to be highly co-linear with two regions of soybean, chromosome 9 and chromosome 15 (Figure 3). The syntenic region in soybean chromosome 9 extended from soybean locus Glyma09g02100 to Glyma09g02560 which corresponded to 17.14 cM to 19.04 cM of the *Fot3-1* locus on the cowpea consensus genetic map (Table 3). The syntenic region was scanned for known disease resistance genes on the soybean genome browser (http://www.phytozome.org) where two soybean disease resistance genes were observed. Soybean locus Glyma09g02210 was flanked by orthologous soybean genes to EST-derived SNP markers 1_1211 and 1_1484 and was annotated as a leucine-rich repeat (LRR) serine/threonine protein kinase (Table 3). Glyma09g02420 was flanked by SNP markers 1_0860 and 1_1107 and was annotated as a disease resistance protein of the NBS-LRR class (Table 3). The *Fot3-1* syntenic locus in soybean chromosome 15 extended from soybean locus Glyma15g12830 to Glyma15g13470 which corresponded to 17.14 cM to 19.04 cM of the *Fot3-1* locus on the cowpea consensus genetic map (Table 3). The syntenic region of soybean chromosome 15 was scanned and four LRR genes were observed, Glyma15g13100, Glyma15g13290, Glyma15g13300 and Glyma15g13310 (Table 3). Glyma15g13100 was flanked by orthologous soybean genes to SNP markers 1_1077 and 1_1484 and was annotated as a LRR serine/threonine protein kinase (Table 3). Soybean loci Glyma15g13290, Glyma15g13300 and Glyma15g13310 were identified between orthologous soybean genes to markers 1_0860 and 1_1212 (Table 3). Glyma15g13290 and Glyma15g13300 were annotated as disease resistance proteins of the NBS-LRR class while Glyma15g13310 was annotated as an LRR protein (Table 3). Due to the high co-linearity of gene order between cowpea and soybean at the two syntenic loci, the observed soybean disease resistance genes were considered as orthologous candidate genes for the *Fot3-1* locus. Soybean is the closest related legume model species to cowpea and both are members of the economically important warm season Phaseoleae clade [12]. The ability to use the sequenced soybean genome as a means to identify candidate genes within syntenic regions in cowpea exhibits the utility of these closely related legumes.

**Table 3:** Synteny of *Fot3-1* with *Glycine max* chromosomes 9 and 15.

*G. max* chromosome	*G. max* locus	Phytozome annotation	Cowpea locus	LG	cM
9	Glyma09g02100	Aspartyl protease	1_1363	6	17.14
9	Glyma09g02130	Sodium hydrogen exchanger	1_0897	6	17.40
9	Glyma09g02160	ENDO-1,4-BETA-GLUCANASE	1_1434	10	45.22
9	Glyma09g02210	Leucine-rich repeat serine/threonine protein kinase	N/A	N/A	N/A
9	Glyma09g02290	Protein of unknown function	1_1484	6	17.88
9	Glyma09g02310	Vesicle-associated membrane protein	1_0860	6	17.82
9	Glyma09g02420	Disease resistance protein (NBS-LRR)	N/A	N/A	N/A
9	Glyma09g02450	Cysteine desulfurylase	1_1107	6	18.31
9	Glyma09g02560	Glycolipid transfer	1_0704	6	19.04
15	Glyma15g12830	DNA-directed RNA polymerase	1_0895	6	17.14
15	Glyma15g13000	Aspartyl protease	1_1363	6	17.14
15	Glyma15g13030	Sodium/hydrogen exchanger	1_0897	6	17.40
15	Glyma15g13080	Glycosyl hydrolase family 9	1_1077	6	17.14
15	Glyma15g13100	Leucine-rich repeat serine/threonine protein kinase	N/A	N/A	N/A
15	Glyma15g13210	Protein of unknown function	1_1484	6	17.88
15	Glyma15g13220	Vesicle-associated membrane protein	1_0860	6	17.82
15	Glyma15g13290	Disease resistance protein (NBS-LRR)	N/A	N/A	N/A
15	Glyma15g13300	Disease resistance protein (NBS-LRR)	N/A	N/A	N/A
15	Glyma15g13310	Leucine-rich repeat protein	N/A	N/A	N/A
15	Glyma15g13330	No functional annotation	1_1212	Not mapped	Not mapped
15	Glyma15g13470	Glycolipid transporter activity	1_0704	6	19.04

**Figure 4:**
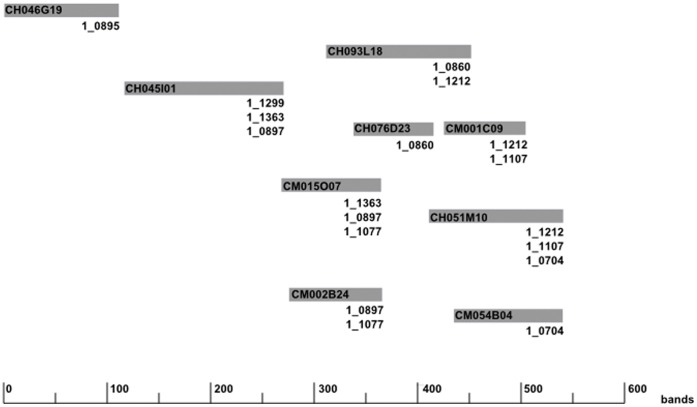
Cowpea BAC contig250 which harbors *Fot3-1*. BAC contig250 consists of 46 BAC clones. Nine BAC clones in the minimum tiling path (MTP) were previously identified as harboring SNP markers and are currently shown. The *Fot3-1* locus spans three overlapping BAC clones, CH093L18, CM001C09 and CH051M10. However, since CH093L18 and CH051M10 overlap the total length of BAC clone CM001C09, *Fot3-1* was narrowed to two overlapping BAC clones which span an approximate total length of 375,560 bp of the total contig length of 885,600 bp. The BAC clones which have been identified with SNP markers are labeled as such. The bar graph at the bottom of the figure represents number of fingerprinting bands.

The cowpea physical map (http://phymap.ucdavis.edu/cowpea) which has been partially anchored to the cowpea consensus genetic map via EST-derived SNP markers was used to identify BAC clones that span the physical region of *Fot3-1.* The most significant markers identified in the biparental mapping study and closely linked markers from the cowpea consensus genetic map identified BAC contig250 as spanning the most significant region of *Fot3-1* (Table 1). The length of the contig is estimated at 885,600 bp (540 nonrepeated fingerprint bands) and consists of 46 BAC clones, 21 of which have BAC-end sequences (BES) available. Nine BAC clones from contig 250 were identified as harboring SNP markers (Figure 4, Table 1). SNP marker 1_1107 was identified on two overlapping BAC clones, CH051M10 and CM001C09 (Figure 4, Table 1). Markers 1_0860 and 1_0704 which are closely flanking markers to 1_1107 on the cowpea consensus genetic map also were found to flank 1_1107 on the physical map (Figure 4, Table 1). 1_0860 was identified on BAC clones CH093L18 and CH076D23 (Figure 4, Table 1). 1_0704 was identified sharing BAC clone CH051M10 with 1_1107, and was also identified on BAC clone CM054B04 (Figure 4, Table 1). Marker 1_1212 which could not be placed on the cowpea consensus genetic map was identified sharing two BAC clones with 1_1107, CH093L18 and CH051M10 (Figure 4). SNP marker 1_1212 was also identified on BAC clone CM001C09, which it shares with marker 1_0860 (Figure 4). Marker 1_1484, which flanks 1_1107 on the cowpea consensus genetic map, was not identified on the cowpea physical map (Table 1). The significant region of the *Fot3-1* locus spanned three overlapping BAC clones, CH093L18, CM001C09 and CH051M10 (Figure 4). However, since CH093L18 and CH051M10 overlap the total length of BAC clone CM001C09, *Fot3-1* was narrowed to two overlapping BAC clones which span an approximate total length of 375,560 bp (Figure 4).

The two BAC clones, CH093L18 and CH051M10, which overlap the significant region of the *Fot3-1* locus were sequenced to identify cowpea candidate genes. The BAC clone sequences were assembled using Velvet software [13]. Cowpea BAC clone CH051M10 which harbored SNP markers 1_1212, 1_1107 and 1_0704, was assembled and resulted in seventy-four contigs with an approximate length of 188,000 to 203,000 bp, which matched the expected size of the BAC clone including the vector (File S2). BAC clone CH051M10 was BLASTed with EST sequences from which SNP markers 1_1212, 1_1107 and 1_0704 were derived to confirm that the markers were present and to assure the quality of the sequence assembly; all three SNP sequences were identified (File S3). The BES of CH051M10 was also identified after BLASTing to the assembled sequence (File S3). The orthologous soybean candidate disease resistance genes were BLASTed to the BAC clone CH051M10 sequences, however, no orthologous cowpea genes were identified which eliminated the BAC as a candidate for harboring the *Fot3-1* gene (File S4).

**Table 4:** Cowpea BAC clone CH093L18 sequences annotated using *Glycine max* BLAST results.

Cowpea sequence	*G. max* locus	*G. max* e-score	Phytozome annotation
NODE_5	Glyma09g02350	3e-083	GDP-fucose protein O-fucosyltransferase
NODE_7	Glyma15g13210	1e-158	APOPTOSIS INHIBITOR 5-RELATED
NODE_10	Glyma09g02340	1e-129	RING/U-box superfamily protein
NODE_11	Glyma09g02310	9e-037	Vesicle-associated membrane protein 721
NODE_13	Glyma08g32320	3e-005	Reverse transcriptase
NODE_14	Glyma02g12430	1e-104	Translation initiation factor 2C
NODE_15	Glyma15g00440	1e-165	SWIM zinc finger
NODE_18	Glyma15g00440	1e-171	SWIM zinc finger
NODE_19	Glyma13g19430	4e-052	Actin depolymerizing factor 1
NODE_20	Glyma09g02280	4e-063	Magnesium transporter CorA-like family protein
NODE_22	Glyma09g02350	8e-099	GDP-fucose protein O-fucosyltransferase
NODE_24	Glyma09g02310	2e-020	Vesicle-associated membrane protein
NODE_25	Glyma15g13120	1e-155	NAD dependent epimerase/dehydratase
NODE_28	Glyma15g13220	5e-016	Vesicle-associated membrane protein 726
NODE_29	Glyma09g02310	3e-009	Synaptobrevin-related protein 1
NODE_32	Glyma02g42330	3e-078	Pleckstrin homology (PH) domain superfamily protein
NODE_33	Glyma09g02350	3e-023	GDP-fucose protein O-fucosyltransferase
NODE_41	Glyma09g02350	5e-009	GDP-fucose protein O-fucosyltransferase
NODE_50	Glyma09g02210	3e-032	Leucine-rich repeat serine/threonine protein kinase
NODE_52	Glyma15g13190	5e-060	SNARE-like superfamily protein
NODE_54	Glyma09g02350	5e-025	O-fucosyltransferase family protein
NODE_56	Glyma15g13250	1e-008	GDP-fucose protein O-fucosyltransferase
NODE_57	Glyma08g34790	7e-010	Leucine-rich repeat serine/threonine protein kinase
NODE_65	Glyma09g02210	3e-012	Leucine-rich repeat serine/threonine protein kinase
NODE_104	Glyma07g40100	9e-008	Leucine-rich repeat serine/threonine protein kinase

The candidate BAC clone CH093L18 which harbors SNP markers 1_0860 and 1_1212 was also sequenced and the assembly resulted in 127 contigs with an estimated length of 184,856 bp (File S5). The EST sequences from which SNP markers 1_0860 and 1_1212 were derived were BLASTed to the assembled BAC clone CH093L18 to confirm their presence and the quality of assembly; both SNPs were identified (File S6). The six soybean candidate genes were BLASTed to CH093L18 to possibly identify orthologous cowpea candidate genes. Glyma09g02210 was the only soybean gene which returned a high similarity with several nodes of the cowpea BAC clone (Table 4). The assembled sequences of BAC clone CH093L18 were then BLASTed to the soybean genome to determine gene annotations for the entire clone. It appeared that there were twenty-five putative cowpea genes on BAC clone CH093L18 and that the only disease resistant-type genes were NODES 50, 57, 65 and 104 which were annotated as leucine-rich repeat serine/threonine protein kinases (Table 4). The *Fot3-1* resistance locus was narrowed to BAC clone CH093L18 and leucine-rich repeat serine/threonine protein kinases were identified as the cowpea candidate gene for *Fot3-1*.

**Figure 5:**
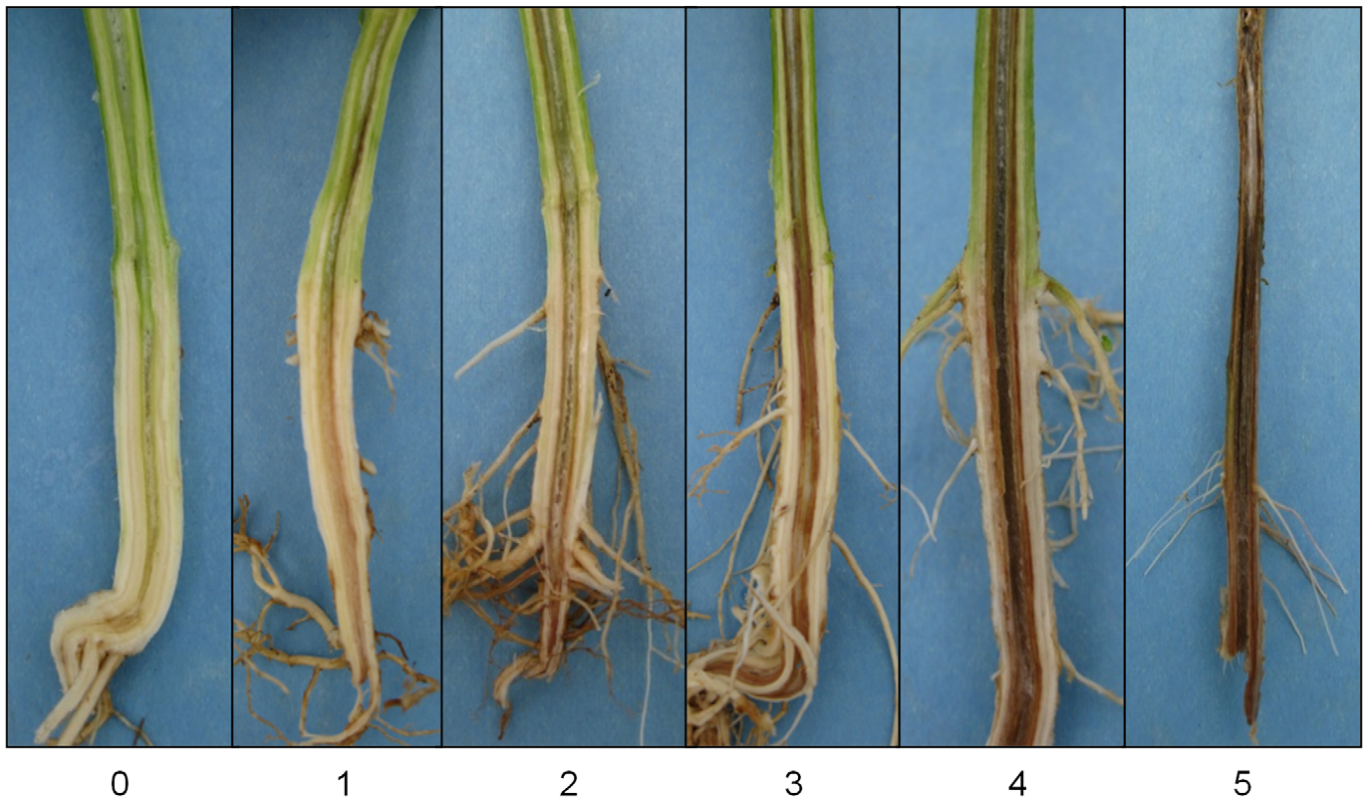
*Fusarium oxysporum* f.sp. *tracheiphilum* phenotyping for vascular discoloration symptoms. The severity of the vascular discoloration disease symptom was evaluated on a zero to five rating scale. A rating of zero indicated a healthy plant with no signs of disease, 1 indicated approximately 10% of the plant showed disease symptoms, 2 indicated approximately 25% of the plant showed disease symptoms, 3 indicated approximately 50% of the plant showed disease symptoms, 4 indicated approximately 75% of the plant showed symptoms and 5 indicated 100% of the plant showed disease symptoms. Five replicates per line were evaluated individually then averaged to determine the disease severity for each RIL.

The soybean candidate disease resistance gene, Glyma09g02210, was BLASTed to the cowpea genome vs. 0.02 to identify candidate genomic sequences for *Fot3-1*. The BLASTn search for the genomic and cDNA sequence of Glyma09g02210 returned a high alignment with scaffold 17795 with e-score values of e-155 and e-147, respectively (File S7). The sequences for scaffold 17795 were then BLASTed back to BAC clone CH093L18 to determine which NODE of the assembly had the highest similarity; NODE 50 returned a perfect alignment with e-score value of 0.0 (File S8). We concluded that NODE 50 on BAC clone CH093L18 was the best candidate cowpea gene for the *Fot3-1* locus and that scaffold 17795 may be the cowpea ortholog to soybean Glyma09g02210.

After determining that *Fot3-1* was located on cowpea BAC clone CH093L18, the physical distance of *Fot3-1* was compared to the cowpea consensus genetic map. The marker-trait association analysis delimited *Fot3-1* to a 1.16 cM region as determined by flanking SNP markers 1_1484 and 1_0704 to 1_1107 (Figure 2, Table 1). Since *Fot3-1* was located on BAC clone CH093L18 which housed SNP markers 1_0860 and 1_1212 (Figure 4); correspondingly, *Fot3-1* was narrowed to a 0.5 cM region on the cowpea consensus genetic map, flanked by SNP markers 1_0860 (17.82 cM position) and 1_1107 (18.31 cM position) since 1_1212 was not positioned on the cowpea consensus genetic map (Table 1).

The cowpea genome size is estimated at 630 Mb [14]. The cowpea consensus genetic map vs.3 [15] estimated the total genetic distance as 680 cM which provides an estimated mean genetic to physical distance ratio of 1.1 cM per Mb. The *Fot3-1* BAC clone CH093L18 is approximately 232,880 bp using the cowpea physical map estimates (http://phymap.ucdavis.edu/cowpea). Therefore, the BAC clone carrying the *Fot3-1* locus and flanking markers at a distance of 0.5 cM has at least two times the mean genetic to physical distance, suggesting that the *Fot3-1* gene resides in a relatively recombination-active region of the cowpea genome. This is fortuitous in the context of resistance gene introgression because the higher recombination rate means a decreased likelihood of deleterious genes being co-introgressed by linkage drag. It also highlights the value of eventually identifying the actual *Fot3-1* gene in order to have a “perfect marker” that will not segregate from the trait.

## Discussion

In this study, we report the identification of the *Fot3-1* locus which confers resistance to Fot race 3 in cowpea. By utilizing the integrated cowpea genomic resources, the *Fot3-1* locus was narrowed to a single BAC clone CH093L18, which identified four leucine-rich repeat serine/threonine protein kinases as candidate genes for *Fot3-1*.

Typically, resistance to Fusarium has been shown to be a dominant and monogenic trait [16], [17], [18], [19], [20] which fits the gene-for-gene hypothesis whereby pathogen and host express complementary dominant genes [21]. The alteration or loss to either the host’s resistance gene or pathogen’s avirulence gene leads to disease [21]. The majority of disease resistance genes are classified as having an NBS-LRR motif which has been further sub-divided by their difference at the N- terminus, either having homology with the TIR domain (TIR-NBS-LRR) [22], [23] or a coiled-coil motif (CC-NBS-LRR or non TIR-NBS-LRR) [23]. Currently, two genes have been cloned which confer resistance to *F*. *oxysporum*, *I-2* and *Fom-2* [24], [25]. The *I-2* locus, which confers resistance to *F. oxysporum* f.sp. *lycopersici* (Fol) race 2 in tomato was determined to be a CC-NBS-LRR disease resistance gene [24]. The *Fom-2* locus, which confers resistance to *F. oxysporum* f.sp *melonis* (Fom) in melon was also identified as a CC-NBS-LRR gene [25].

Although the majority of cloned R genes have the conserved NBS-LRR structure, there are several disease resistance genes identified as belonging to the receptor-like kinase (RLK) family. RLKs are proteins that span the plasma membrane, recognizing and responding to extracellular signals [26]. The majority of RLK have serine/threonine kinases and LRR motifs [27]. The receptor-like cytoplasmic kinase (RLCK) disease resistance genes include *PBS1*, *Pti* and *Pto* [28]. *PBS1* confers resistance against *Pseudomonas syringae* pv *phaseolicola* in Arabidopsis [29]. *Pti* and *Pto* both confer resistance to the bacterium *Pseudomonas syringae* pv *tomato* [30], [31]. *Xa21* is a LRR RLK and confers resistance against *Xanthomonas campestris* pv *oryzae* in rice [32]. *Lrk10* which confers resistance to the fungus, *Puccinia recondite* in wheat was also determined to be a serine/threonine protein kinase [33]. The *I-3* locus which confers resistance to *F. oxysporum* f.sp. *lycopersi* race 3 in tomato, was determined to be positioned within a large cluster of S-locus receptor-like kinases (SRLK) [34]. Interestingly, we recently identified TIR-NBS-LRR proteins and leucine-rich repeat serine/threonine protein kinases in the *Fot4-1* and *Fot4-2* syntenic regions of soybean (unpublished data). *Fot4-1* and *Fot4-2* confer resistance to Fot race 4 in cowpea (unpublished data). It may be possible that leucine-rich repeat serine/threonine protein kinases are the R genes conferring resistance in the cowpea-Fusarium pathovar system.

A practical outcome of this study is the development of molecular markers closely linked to the *Fot3-1* locus. These markers can be used in marker-assisted breeding to optimize cowpea genetic improvement via different strategies including pedigree backcrossing and marker-assisted recurrent selection. These approaches should expedite variety development by at least halving the current traditional breeding selection process which relies on time-consuming and costly phenotyping. The identification of the Fot race 3 resistance gene would provide ‘perfect markers’ and further improve marker-assisted breeding efficiency.

Future goals include functional analysis of *Fot3-1* candidate genes to define the genetic resistance determinant. Identifying the *Fot3-1* gene will enhance our understanding of resistance to Fusarium as well as broaden our knowledge of resistance genes within the legume family.

## Materials and Methods

Resistance to Fot race 3 was tested on a RIL population which was developed by an intraspecific cross between cultivar California Blackeye 27 (CB27) and ‘C93W-24-125B-1’. Each of the 90 lines was advanced by single seed descent to the F_10_ generation. CB27 is a cultivar which was bred for resistance to *F. oxysporum* f.sp. *tracheiphilum* races 3 and 4 [8]. C93W-24-125B is a breeding line from Cameroon and is highly susceptible to Fot race 3 [35], [36]. These materials were available from the University of California Riverside cowpea germplasm collection.

Two strains of Fot race 3, which were isolated previously from infected cowpea plants in the San Joaquin Valley, California, were used for inoculum cultures (unpublished data, Shirley Smith). Individual strains were developed from single spore lines. Isolates were dried and stored on sterile potato dextrose agar (PDA) plates at −80°C. 1-cm^2^ plugs were cut from frozen Fusarium-containing PDA plates and transferred aseptically to flasks containing 500 ml of potato-dextrose broth, then incubated in a shaker at 27°C and 30 rpm under lighted conditions for three days. The liquid culture was strained through four layers of cheesecloth to eliminate mycelium, followed by adjustment of the spore concentration to 1.0×10^6^ microconidia per ml using a hemocytometer. Greenhouse experiments were conducted using a modified root-dip inoculation method as previously described [37]. Ten greenhouse grown seeds per line were planted in seeding trays filled with vermiculite and watered daily for one week. After one week, five seedlings per line were gently uprooted and half of the root system was clipped and then dipped for one minute into suspended inoculum. Inoculated seedlings were transplanted into one gallon pots, randomized on benches and watered daily. Greenhouse day temperatures were set to 28°C and night temperatures set to 16°C.

Plants were evaluated five weeks post inoculation for Fusarium disease symptoms. The wilting/stunting phenotype was evaluated by approximating the percentage of wilting or stunting on the entire plant. The vascular discoloration phenotype was evaluated by uprooting the entire plant, then slicing the stem vertically to evaluate the extent of the disease symptoms (Figure 5). The severity of the disease was evaluated on a zero to five rating scale for the wilting/stunting and vascular discoloration phenotypes. A score of zero indicated a healthy plant with no signs of disease, 1 =  approximately 10% of the plant showing symptoms of disease, 2 =  approximately 25% of the plant showing symptoms of disease, 3 =  approximately 50% of the plant showing symptoms, 4 =  approximately 75% of the plant showing symptoms and 5 = 100% of the plant showing disease symptoms. Five replicates per line were evaluated individually then averaged to determine the disease severity for each RIL.

The California Blackeye 27×24-125B-1 population and genotypes CB27, CB46, Iron Clay, SH49-10-4-1-1, SH50-17-9-1-1 (also known as California Blackeye No. 50), SH50-7-9-2, IT93K-503-1, 24-125B-1, CB5, Bambey 21, IT82E-18/Big Buff and IT84S-2049 were genotyped at the F_8_ generation or above using biallelic SNP markers from the 1536 Illumina GoldenGate Assay as previously described in Muchero, et al. (2009).

A SNP genetic map for the California Blackeye 27×24-125B-1 population was created previously and is included in both cowpea consensus genetic map vs.2 [10] and vs. 3 [15]. The map was generated using 339 SNP markers and 90 individuals and consisted of sixteen linkage groups and spans approximately 600 cM total distance [15]. The cowpea consensus genetic map vs. 3 [15] was used for this study which is an updated version of the Muchero, et al. (2009) map. The vs. 3 map was developed using ten RIL populations and two breeding populations which increased the marker density and improved the marker order [15]. The vs. 3 consensus genetic map is 680 cM in length and contains 1043 markers which is an addition of 115 markers and an average 0.65 cM between markers [15]. The current SNP-based cowpea linkage map is included in a publicly available browser called HarvEST:Cowpea, which can be downloaded as a Windows software from http://harvest.ucr.edu or viewed online at www.harvest-web.org.

Resistance to Fot race 3 was mapped using the CB27 × 24-125B-1 genetic map and greenhouse inoculation datasets which were comprised of wilting/stunting and vascular discoloration phenotypes. Kruskal-Wallis and Interval Mapping analysis packages of MapQTL 5.0 software were used to conduct the bi-parental mapping [38]. A locus was considered significant if the same locus was identified using both phenotypic ratings and if the statistical tests for the markers met significance thresholds for both Kruskal-Wallis and Interval Mapping analyses. A significance threshold was set to 0.05 for Kruskal-Wallis analysis and LOD thresholds for the Interval Mapping analysis were calculated using 1000 permutations at the 0.05 significance level. A 95% confidence interval was used to determine the span of the locus using 1-LOD and 2-LOD to determine left and right margins. Results were visualized using MapChart 2.2 software [39].

Synteny was examined between cowpea and *G. max* using EST-derived SNP markers previously BLASTed and aligned to the sequenced genomes as described previously [10]. Syntenic relationships between the cowpea, soybean, *Medicago truncatula* and *Arabidopsis thaliana* can be examined in HarvEST:Cowpea database (http://harvest.ucr.edu). Syntenic maps were drawn using HarvEST:Cowpea using a cut-off e-score value of -10, with a minimum number of 13 lines drawn per linkage group. Due to limited resolution in the software images, not all markers are presented in the screenshot images output from Harvest:Cowpea. In order to view each individual marker, the linkage group must be magnified in the HarvEST:Cowpea database.

The cowpea physical map (http://phymap.ucdavis.edu/cowpea) was developed in work to be described elsewhere using an advanced African breeding line IT93K-399-35 and two BAC clone libraries developed with restriction enzymes *Hind*III and *Mbo*I (Amplicon Express, Pullman, WA). Contigs were assembled using the snapshot method of DNA fingerprinting [40] and was completed at the University of California, Davis by Ming Cheng Luo. The length of the BAC clones was estimated by multiplying the number of unique bands generated from the fingerprinting assay by 1640 bp (personal communication, Ming Cheng Luo).

BAC clones CH051M10 and CH093L18 were sequenced using an Illumina GA_II_ or HiSeq 2000 sequencer, respectively, at the Institute of Integrative Genomics Biology, University of California, Riverside. BAC clones were purified using a QIAGEN 96 prep kit following manufacturer’s instructions (Valencia, CA). Purified BAC clones were sheared using a Diogenode Bioruptor UCD-200 (Liege, Belgium) for 14 minutes at the maximum setting, alternating on and off for 30 seconds. Fragments ranging from 300-500 bases in length were visualized and excised from a 1% precast E-gel® (Invitrogen, Carlsbad, CA). BAC clone fragments were prepared for sequencing using Illumina’s Paired End DNA Sample Prep kit following manufacturer’s instructions. A QIAquick PCR Purification kit was used in between amplification steps (QIAGEN, Valencia, CA). Sequences from CH051M10 were generated as 36-base single-end reads from a single sample on an Illumina GA_II_ instrument. CH093L18 sequences were generated as 100-base paired-end reads within a 14-sample multiplex in one lane on an Illumina HiSeq 2000 instrument. BAC clone sequences were first filtered to remove *E. coli* sequences then assembled using Velvet software [13] using a range of k-mer lengths from 19 to 35 to identify an optimal assembly considering the estimated depth of coverage, number of nodes, N50 and maximum node length. The optimum assembly of CH051M10 was obtained using k-mer size 25 (N50 = 6,384). The optimum assembly of CH093L18 was obtained using k-mer size 27 (N50 = 7,717). A NODE is defined as a sequence or contig which can be consistently reconstructed using the sequencing reads [13], [41]. All sequence data is publicly available via the Harvest:Cowpea database (www.harvest.ucr.edu) and version 0.02 of the assembled cowpea genome (www.harvest-blast.org).

Cowpea genome version 0.02 which contained approximately 200 Mb of assembled scaffolds and contigs covered about 97% of previously identified cowpea genes (UCR cowpea group, unpublished) is available for BLAST searches and sequence retrieval (www.harvest-blast.org).

## Supporting Information

File S1 **SNP Marker 1_1107 sequence.** cDNA sequence of P12 assembly unigene 12265 which is housed in Harvest: Cowpea database (http://harvest.ucr.edu). The adenine/guanine SNP is located at position 693, parenthesized and in bold.
(DOCX)

File S2 **FASTA file for cowpea BAC clone CH051M10.**
(TXT)

File S3 **BLAST of cowpea SNP markers and BES to cowpea BAC clone CH051M10.**
(DOCX)

File S4 **Soybean candidate genes BLASTed to cowpea BAC clone CH051M10.**
(DOCX)

File S5 **FASTA file for cowpea BAC clone CH093L18.**
(TXT)

File S6 **Cowpea SNP markers BLASTed to cowpea BAC clone CH093L18.**
(DOCX)

File S7 **Soybean candidate genes BLASTed to the cowpea genome.**
(DOCX)

File S8 **Cowpea genomic sequences BLASTed to BAC clone CH093L18.**
(DOCX)
