# Supplementary material for: Genetic and Physical Mapping of Candidate Genes for Resistance to Fusarium oxysporum f.sp. tracheiphilum Race 3 in Cowpea [Vigna unguiculata (L.) Walp]
Source: PLoS One. 2012 Jul 31;7(7):e41600. doi: 10.1371/journal.pone.0041600 (PMC3409238; doi:10.1371/journal.pone.0041600)
Supplement: File S1 — SNP Marker 1_1107 sequence. cDNA sequence of P12 assembly unigene 12265 which is housed in Harvest: Cowpea database (http://harvest.ucr.edu). The adenine/guanine SNP is located at position 693, parenthesized and in bold. (DOCX) [file pone.0041600.s001.docx]

S1. SNP marker 1_1107 EST sequence and SNP position.

GAGCAAATTGAAAGATATGTTCTCACGGAAGACCAAAATAGTAGTCGCTCATCATGTTTCAAATGTGCTTGCTTCTGTCCTTCCTATTAGAGATATTGCACAATGGGCACATGATGTTGGAGCAAAAGTTCTTGTATGCTTGTCAGAGTGTTCCACACATGGTGGTTGATGTCCAGAGCCTTAATGTTGATTTTCTTGTTGCTTCTTCTCACAAGATGTGTGGGCCTACGGGAATTGGATTCTTATATGGTAAAATAGACCTCTTGTCTTCCATGCCTCCATTTTTAGGTGGTGGTGAAATGATTTCTGATGTATATCTTGATCATTCAACTTATGCCGAACCTCCTTCCAGATTTGAGGCTGGAACACCAGCTATTGGGGAAGCAATTGGTTTAGGAGCAGCAATTGATTACTTATCTGGGATTGGTATGCAAACTATACATGATTATGAGGTGGAGCTTGGTAGTTATCTGTACGAAAGGCTTCTTTCAGTCCCAAATATTCGCATCTATGGGCCAGCACCTTCAGAAAATGTTCAACGAGCAGCTCTTTGTTCTTTCAATGTTGAGAATTTGCATCCCACTGATCTTGCAACATTTCTGGACCAACAGCATGGAGTGGCTATCAGATCAGGTCACCATTGTGCCCAACCCCTCCATCGCTTCTTAGGAGTCAGCTCAAGTGCACGCGCC**(A/G)**GTCTCTACTTCTACAACACAAAGGAAGATGTGGACTACTTTATCCATGCCCTCAACGACACAGTCAACTTTTTCAACTCATTCAAGTAACCAGAATGTATTTTAATGTATATTAAATTTTGTTTATACGCCAATGAGAGGGTTGTCTTAGTTGGTAGGAAAGCTGCGTCAATGAAATGTTCTTGAATTTCATTCCTTCTATTGATGTCAATGGTAGGAACTAGGCATCCATTAATTGCAGTATTGAAACCTATCTACAGCTGAACTTTTATGCATAAAAAGAATGCCCATAAGCATTTTAATTAAAAAAAAAAAAAAAGTTGGAAGTTGAATGTTTTATCCATTTTTACTTTTTGATGGAATAAAAAAAAAAAAAAAAA
