# Supplementary material for: Genetic and Physical Mapping of Candidate Genes for Resistance to Fusarium oxysporum f.sp. tracheiphilum Race 3 in Cowpea [Vigna unguiculata (L.) Walp]
Source: PLoS One. 2012 Jul 31;7(7):e41600. doi: 10.1371/journal.pone.0041600 (PMC3409238; doi:10.1371/journal.pone.0041600)
Supplement: File S3 — BLAST of cowpea SNP markers and BES to cowpea BAC clone CH051M10. (DOCX) [file pone.0041600.s003.docx]

| S3. BLAST of cowpea SNP markers and BES to cowpea BAC clone CH051M10. | | | |
| --- | --- | --- | --- |
| Sequence of SNP or BES | Sequence position | Bits | e-score |
| 1_0704 | NODE_16 | 549 | e-158 |
| 1_1212 | NODE_30 | 460 | 3-131 |
| 1_1107 | NODE_18 | 737 | 0.0 |
| BES of CH051M10 | NODE_3 | 1548 | 0.0 |

SNP = single nucleotide polymorphism, BES = Bacterial Artificial Chromosome-end sequence
