# Supplementary material for: Genetic and Physical Mapping of Candidate Genes for Resistance to Fusarium oxysporum f.sp. tracheiphilum Race 3 in Cowpea [Vigna unguiculata (L.) Walp]
Source: PLoS One. 2012 Jul 31;7(7):e41600. doi: 10.1371/journal.pone.0041600 (PMC3409238; doi:10.1371/journal.pone.0041600)
Supplement: File S4 — Soybean candidate genes BLASTed to cowpea BAC clone CH051M10. (DOCX) [file pone.0041600.s004.docx]

| S4. *Glycine max* candidate genes BLAST to cowpea BAC clone CH051M10. | | | | | | |
| --- | --- | --- | --- | --- | --- | --- |
| *G. max* locus | tBLASTn | Bits | e-score | BLASTn | Bits | e-score |
| Glyma09g02210 | NODE_19 | 23 | 4.2 | NODE_4 | 30 | 0.290 |
| Glyma09g02420 | NODE_22 | 23 | 4.6 | NODE_16 | 30 | 0.400 |
| Glyma15g13100 | NODE_31 | 26 | 0.72 | NODE_34 | 30 | 0.420 |
| Glyma15g13290 | NODE_21 | 23 | 7.4 | NODE_50 | 32 | 0.098 |
| Glyma15g13300 | NODE_18 | 25 | 2 | NODE_16 | 30 | 0.400 |
| Glyma15g13310 | NODE_11 | 23 | 1.9 | NODE_12 | 30 | 0.180 |
