# Supplementary material for: Genetic and Physical Mapping of Candidate Genes for Resistance to Fusarium oxysporum f.sp. tracheiphilum Race 3 in Cowpea [Vigna unguiculata (L.) Walp]
Source: PLoS One. 2012 Jul 31;7(7):e41600. doi: 10.1371/journal.pone.0041600 (PMC3409238; doi:10.1371/journal.pone.0041600)
Supplement: File S6 — Cowpea SNP markers BLASTed to cowpea BAC clone CH093L18. (DOCX) [file pone.0041600.s006.docx]

| S6. Cowpea SNP markers BLAST to cowpea BAC clone CH093L18. | | | |
| --- | --- | --- | --- |
| Cowpea locus | Sequence position | Bits | e-score |
| 1_0860 | NODE_24 | 827 | 0 |
| 1_1212 | NODE_3 | 460 | e-131 |
