# Supplementary material for: Genetic and Physical Mapping of Candidate Genes for Resistance to Fusarium oxysporum f.sp. tracheiphilum Race 3 in Cowpea [Vigna unguiculata (L.) Walp]
Source: PLoS One. 2012 Jul 31;7(7):e41600. doi: 10.1371/journal.pone.0041600 (PMC3409238; doi:10.1371/journal.pone.0041600)
Supplement: File S7 — Soybean candidate genes BLASTed to the cowpea genome. (DOCX) [file pone.0041600.s007.docx]

| S7. *Glycine max* candidate gene BLAST to the cowpea genome. | | | | | | |
| --- | --- | --- | --- | --- | --- | --- |
| *Glycine max* locus | BLASTn (genomic) | Bits | e-score | BLASTn (cDNA) | Bits | e-score |
| Glyma09g02210 | scaffold 17795 | 553 | e-155 | scaffold 17795 | 523 | e-147 |
