# Supplementary material for: Genetic and Physical Mapping of Candidate Genes for Resistance to Fusarium oxysporum f.sp. tracheiphilum Race 3 in Cowpea [Vigna unguiculata (L.) Walp]
Source: PLoS One. 2012 Jul 31;7(7):e41600. doi: 10.1371/journal.pone.0041600 (PMC3409238; doi:10.1371/journal.pone.0041600)
Supplement: File S8 — Cowpea genomic sequences BLASTed to BAC clone CH093L18. (DOCX) [file pone.0041600.s008.docx]

| S8. Cowpea genomic sequences BLAST to BAC clone CH093L18. | | | |
| --- | --- | --- | --- |
| Cowpea genomic sequences | Bits | e-score | Sequence position |
| scaffold 17795 | 2343 | 0.0 | NODE 50 |
